# Dialogical Self in a Complex World: The Need for Bridging Theories

**DOI:** 10.5964/ejop.v11i1.917

**Published:** 2015-02-27

**Authors:** Hubert J. M. Hermans

**Affiliations:** a Radboud University of Nijmegen, Nijmegen, The Netherlands

Some decades ago, I lived, like many of my colleagues, in the illusion that the word was made up of two realities that were essentially different: a physical material world governed by deterministic laws and a psychological or ‘mental’ reality that centered around human beings who were blessed with a free will that liberated them from determination and predictability. Gradually, however, I noticed that this dualistic perception was untenable as I became aware of the work of physicists—quantum physics and chaos theory in particular—who had created room for *indeterminism* and psychologists who acknowledged that even conscious decisions are, at least partly, *determined* by non-conscious processes preceding conscious awareness.

In the last decades, we notice that psychology is more and more ‘infiltrated’ by the neurosciences, first in the form of cognitive neuroscience, but later in the shape of social neuroscience, affective neuroscience, and even cultural neuroscience. Recent trends in the neuroscientific domain demonstrate that interest is moving downward, from cortical to sub-cortical levels (from slow conscious thinking to rapid, non-conscious determinants) and from left-brain to right brain functioning (from analytic, step by step analysis to intuitive, imaginative, and holistic evaluation).

What does this mean for the self? Advances in psychology, neurosciences, sociology, and cultural anthropology, all interested in the self from divergent perspectives, result in the view that the self is highly complex as a social, societal, brain-based, and body-based construct. The question is then, how can we make theories extensive and flexible enough to create bridges between the different, highly specialized disciplines and sub-disciplines. I have the strong feeling that, given their limited specialized scope, not any of the existing psychological sub-disciplines is well-equipped to develop a self-theory broad enough to give an adequate answer to the complexities of the self. In my view, if such a theory is to be developed, it should profit from existing knowledge provided by the specialized (sub)-disciplines and, at the same time, to transcend their boundaries to construct a ‘broad picture’ view on the self as it is socially and culturally becoming more multi-faceted and complex as part of a globalizing and boundary-transcending world.

Together with my colleagues, I’m working on Dialogical Self Theory (Hermans & Gieser, 2012) that we have presented as a ‘bridging theory’. Such a theory is neither constructed as a grand theory, pretending to offer an all-comprehensive explanation of human behavior, nor as a mini-theory that is focused on a fairly narrow portion of human functioning. It is also not a theory that aims to combine or integrate two or more existing mini-theories in any synthesizing way. Rather, it is bridging in the sense that a larger diversity of theories, research traditions, and practices meet, or will meet, in order to create new and unexpected linkages. The notion of bridging theory should not suggest that it exists of mediating channels only, linking existing insights or practices without providing an original and new view of the self. Instead, it is a theory with an own identity, scope, and specific conceptual framework. At the same time, the conceptual framework is open enough to different, separated, or even contradictory conceptual systems and practices to provide a platform where they can meet in the service of their mutual enrichment and further development.

Such a theory needs a central concept that is flexible enough to be applied to a broad variety of phenomena and their mutual relationships. This concept, at the heart of the theory, is the ‘*I-*position.’ In this composite term two elements come together: ‘I’ and ‘position.’ Inspired by American pragmatists, like William James and George Herbert Mead, the word ‘I’ refers to a subject-position, a first-person perspective, from which the world and the self is perceived, experienced, and evaluated. The notion of ‘position,’ inspired by Mikhail Bakhtin’s dialogical approach, emphasizes the spatial nature of the self. The self is always somewhere, placed towards other positions in and outside the self and establishing relationships with these other positions. Moreover, dynamic as an *I*-position is, it can take the form of active positioning (e.g., as a critic, as cooperative, as looking for support) but also in the passive form of being positioned by others in social or societal contexts (e.g., as trustworthy or untrustworthy, as normal or abnormal). On its turn, the self is able to respond to these ‘determining’ influences, with an ‘answering’ position, agreeing or disagreeing with the way it is positioned by others. In this ways the process of positioning is an iterative and reciprocal way of participants placing themselves and others as part of social, cultural or societal forms of relationships. Its spatial nature is expressed in the verbs *positioning* and *counter-positioning* while its temporal manifestations are expressed in the form of *positioning* and *repositioning*. As part of a transcendental awareness, the self can *deposition* itself, liberating oneself from being or becoming imprisoned within overly limiting boundaries.

For the understanding of the notion of *I*-position, it is essential to abandon the idea of sharp, razor-like, boundaries between self and other. Certainly, the other has an existence that can be distinguished from the self, but at the same time the other is part of the self, in the form of ‘the-other-in-the-self’. This view is in agreement with James who claimed that the other is part of the extended self and with Bakhtin who considered the other as ‘another I,’ suggesting that the other occupies a subject position in the extended self. Bringing together self and other is also foundational in Martin Buber’s vision who considers I and Thou as a word-pair, suggesting that as components they essentially belong together and resist any separation in the form of isolated essences.

Along these lines, we have proposed a dialogical self as a composite term in which James’s extended self is brought together with Bakhtin’s notion of dialogue. In this way, a between-concept (dialogue) is combined with a within-concept (self) so that a conceptual basis is created for the transportation of the internal constructions of the self to the social and societal world and vice versa. In this way, self and the social environment intrinsically belong together and may question, correct, enrich each other and contribute to each other’s development.

The embodied self and other are situated, localized, and positioned in a physical space in which they are continuously involved in interactions with each other. This physical space is the basis of the metaphorical space of the self in which a dynamic multiplicity of *I*-positions is continuously involved in a variety of dialogical (or monological) relationships. The dominance and power relationships in society at large are reflected in dominance and power relationships in the mini-society of the self. However, the self is not a pure ‘reflection’ of what is taking place outside and it is not simply determined by external factors. On the basis of its own agentic potentials the dialogical self is able to give, in the form of counter-positioning, any answer to the influences coming from outside. Along these lines, the dialogical self has the potential to escape both determinism and self-other dualism.

Contributions from different fields of application reflect the boundary-crossing nature of Dialogical Self Theory as a bridging theory: cultural psychology, educational psychology, philosophy, psychotherapy, personality psychology, psychopathology, developmental psychology, experimental social psychology, career counseling, brain sciences, psychoanalysis, social work, psychodrama, cultural anthropology, religion, literary analysis, philosophy, the psychology of the internet, and the psychology of globalization.

I wrote this editorial, two days after the Charlie Hebdo drama in Paris took place. I realized the enormous power of emotions that spread through the western world as an all-covering flood. I noticed in other people and also in myself how strong the emotional positioning was towards the perpetrators and, in a very different way, towards the victims. At the same time, I realized how important it is not to lose oneself in a war-like mood, but to find, difficult as it is, a counter-position in reason. A productive dialogue between emotional and reasonable positions is needed, particular in this tumultuous period and, certainly, also in its aftermath. I hope that ‘I’m Charlie,’ emerging as a powerful collective *I*-position, will be a source of energy for transcending existing boundaries both in the self and the world.
